# Paxillin promotes the invasion and migration of polyploid giant cancer cells with daughter cells after arsenic trioxide treatment by regulating the expression of cathepsin B/D

**DOI:** 10.7150/jca.115892

**Published:** 2025-07-24

**Authors:** Jiangping Wang, Xiaorui Wang, Na Li, Kai Liu, Shiwu Zhang

**Affiliations:** 1 Department of Pathology, Tianjin Union Medical Center, Tianjin, 300071, China.; 2 Graduate School, Tianjin Medical University, Tianjin, 300070, China.; 3 Department of Research and Education, Tianjin Beichen Hospital, Tianjin, China

**Keywords:** paxillin, CDC42, cathepsin B/D, colorectal cancer, polyploid giant cancer cells

## Abstract

**Purpose:** Polyploid giant cancer cells (PGCCs) play an important role in regulating heterogeneity, growth, and chemotherapy resistance of malignant tumors. Paxillin is a unique cytoskeletal protein and drives persistent migration. In this study, we investigated the molecular mechanism by which paxillin regulates the invasion and migration of PGCCs with daughter cells (PDCs).

**Methods:** We treated HCT116 and LoVo cells with arsenic trioxide (ATO) to induce the formation of PGCCs, and the migration, invasion, and proliferation abilities of PDCs were measured using wound-healing, western blot, immunofluorescence, cell transfection, and dequenching (DQ)-gelatin assays.

**Results:** ATO-induced PDCs had higher invasion, migration, and proliferation ability. Focal adhesion protein paxillin, cytoskeletal protein CDC42, and protease-related protein cathepsin B/D were highly expressed in PDCs. CDC42 promotes the phosphorylation of paxillin by regulating the expression of integrin β1. When phosphorylated at specific tyrosine residues, paxillin plays an important scaffold role in cell adhesion by recruiting structural and signaling molecules (cathepsin B/D) involved in cell movement and migration. Cathepsin B/D can also promote the phosphorylation of paxillin and FAK and promote the invasion and migration of PDCs by degrading the extracellular matrix and inducing cytoskeleton disorders.

**Conclusion:** Paxillin phosphorylation plays an important role in PDCs invasion and migration. Paxillin may be a potential predictor of metastasis and an independent prognostic factor for recurrence and may target phosphorylation to mitigate the impact of chemotherapy-resistant cells on cancer progression, thereby improving patient outcomes.

## Introduction

Colorectal cancer (CRC) is the third most common type of cancer globally and is the second leading cause of deaths attributed to cancer 1. Despite the increasing survival rate, metastatic CRC is still a fatal disease, with a 5-year survival rate of approximately 14%. Although early CRC can be cured by surgery or adjuvant chemotherapy, the presence of metastatic active cells that are resistant to treatment largely determines that the tumor is incurable 2. Therefore, recurrence, metastasis, and drug resistance are still challenges in the treatment of CRC.

Polyploid giant cancer cells (PGCCs) with cancer stem cell characteristics can cause increased resistance to chemotherapy, metastasis, and recurrence, ultimately leading to reduced survival. PGCCs can be formed by nuclear replication or cell fusion and induced by multiple factors, including hypoxic mimic, chemical reagents, radiotherapy, Chinese herbal medicine and viral infections 3, 4. Through temporary dormancy, PGCCs evade attacks on the mitotic mechanism to ensure the survival. PGCCs can exit dormancy after favorable conditions are restored, and generate daughter cells to resume proliferation, leading to the regeneration of more aggressive tumors. This presents a challenge in developing novel treatments for PGCCs with daughter cells (PDCs). Therefore, an emergency need arises to investigate the molecular pathways underlying the augmented invasive and migratory capabilities of PDCs. Previous studies have shown that PDCs exhibit a dynamic and flexible cytoskeleton, thereby endowing them strong migration and invasive capacity. PDCs may mediate collective invasion via Rho kinase or regulate extracellular matrix (ECM) degradation through cathepsin activity 5. Additionally, circulating giant cancer cells in the blood are associated with poor prognosis in various cancer types 6. PDCs are more prevalent at the invasion front and metastatic foci of human serous ovarian cancer 7, 8. In breast cancer and glioma, the number of PDCs increases significantly with increasingly higher malignant tumor grades 9-12. Recurrence, metastasis, and chemoradiation resistance of malignant tumors are closely associated with the number of PGCCs 13. PDCs have stronger invasion and migration abilities than common tumors. Therefore, exploring the molecular mechanisms underlying PDCs, tumor recurrence, metastasis, and drug resistance is important for the development of new therapeutic approaches 14.

Tumor-stromal cell interactions have been identified as a crucial factor promoting drug resistance and the spread of metastasis 15. Although the nuclei of PGCCs are hard, this makes them more prone to deformation. This feature is combined with an increased ability to generate traction, indicating that the cytoskeleton network drives PGCCs to easily invade the surrounding matrix and form a highly invasive subgroup 16. MDA-MB-231 polyploid breast cancer cells have a higher level of actin bundles with longer and thicker stress fibers. These stress fibers establish connections between the cytoskeleton and ECM through focal adhesions, potentially influencing cytoplasmic rigidity directly 17. In addition, PGCCs obtained stabled interphase cytoskeleton and stiffness after treated with paclitaxel, a tubulin stabilizer. Genotoxically-treated PGCCs usually exhibit a dynamic and flexible cytoskeleton, a characteristic that enables PGCCs to circulate efficiently in the blood.

In this study, ATO was used to induce the formation of PGCCs via cell fusion mediated by GCM1/syncytin-1. Syncytin-1, a membrane glycoprotein derived from the human endogenous retrovirus, mediates trophoblast cell fusion and immune response in placental development. The fusion process is highly similar to the formation of PGCCs, suggesting that PGCC may acquire the “embryonic invasion ability” to degrade the ECM 18. The formation of PGCCs is also similar to embryo implantation. In the adriamycin-treated breast cancer model, PGCCs form a structure analogous to a “pregnancy like system” via mitotic slippage 19. The evolutionary conservation of the GCM1/syncytin-1 pathway implies that the invasion of PDCs may share the molecular basis with the maternal-fetal interaction during embryo implantation 20. In PDCs, the GCM1/syncytin-1 pathway is reactivated, which not only mediates cell fusion but also enhances cytoskeletal dynamics and traction through downstream signaling (e.g., Rho kinase and FAK pathway) and collaborates with cathepsin to achieve efficient ECM degradation. This phenomenon of module reuse indicates that the mechanism of tumor invasion is not entirely de novo evolution but rather involves co-opting development-related pathways to rapidly acquire metastasis capabilities. This association not only explains the local mechanism of ECM degradation but also reveals a biological similarity between PGCCs formation and the embryological of cancer.

Paxillin is a multifunctional multidomain adaptor protein that is involved in cell adhesion, diffusion, and migration by recruiting a large number of focal adhesion structural proteins associated with signaling pathways 21. Paxillin is at the intersection of cell adhesion and growth factor regulation of intracellular signaling pathways. The phosphorylated paxillin at tyrosine 118 (Y118) is the main tyrosine phosphorylation site involved in focal contact transition and cell motility 22. Cell division cycle 42 (CDC42), the most metastatic Rho GTPase, is a key regulator of severe paxillin residue phosphorylation after adhesion 5, 23. Cathepsin B can initiate the proteolytic cascade to degrade a range of extracellular matrices and facilitate tumor invasion and migration. However, it is unclear whether they interact with each other. Abnormal expression of cytoskeletal regulatory proteins and remodeling of the ECM can promote tumor development and chemotherapy resistance.

In this research, we investigated the molecular mechanism by which high paxillin phosphorylation in PDCs leads to the formation of daughter cells with enhanced invasive and migratory abilities. CDC42 promotes the phosphorylation of paxillin and degradation of the ECM through cathepsin B/D. Cytoskeletal rearrangement and abnormal cell adhesion are key steps in the metastasis of PDCs.

## Methods

### Cell culture and arsenic trioxide (ATO)-induced PGCC formation

LoVo and HCT116 cell lines were obtained from American Type Culture Collection. Both cell lines were cultured in RPMI-1640 medium supplemented with 10% fetal bovine serum (FBS, Life Technologies, New Zealand) and 100 U/mL penicillin (Gibco, Life Technologies, USA). When the confluency of cells reached 70%, 32 μM ATO (provided by Professor Gao Yu from the Department of Pharmacy, Beijing Institute of Radiological Medicine) was added to the medium with serum for 48 h for HCT116 cells, and for 24 h for LoVo cells. After three treatments, PGCCs accounted for approximately 30% of the total cells, and the remainder were daughter cells of PGCCs.

### Immunofluorescent staining

The cells were seeded in a six-well plate with a coverslip. When the cells grew to the suitable density, they were fixed with 4% paraformaldehyde for 20 min, washed with phosphate-buffered saline (PBS), blocked with 5% bovine serum albumin (BSA) for 2 h, and then incubated overnight with primary antibodies. (Antibody information is shown in Supplementary Table S1). The next day, Alexa Fluor 594 or Alexa Fluor 488 coupled fluorescent secondary antibodies were added, and the nuclei were stained with DAPI reagent.

### Wound-healing assay

A wound-healing assay was used to evaluate cell migration ability. When the cells grew in the six-well plate reached the appropriate confluency, the cell surface was scratched with a sterile pipette tip and the medium was replaced with serum-free medium to continue culturing. Photographs were taken at 0 and 24 h.

### Transwell migration and invasion assay

A transwell migration assay was used to evaluate cell migration ability. A 200 μL cell suspension containing 1×10^5^ cells was inoculated into the upper chamber of the transwell migration chamber, and 600 μL medium containing 20% FBS was added to the lower chamber. After 24-48 h of incubation, the cells were fixed with methanol for 30 min, then stained with 0.1% crystal violet for 30 min, and photographed after mounting.

### Plate colony-formation assay

A colony-formation assay was used to evaluate cell proliferation, and 30, 60, and 120 cells were seeded in 12-well plates. After 1-2 weeks of culture, the cells were fixed with methanol for 30 min, and then stained with 0.1% crystal violet for 30 min to count the number of cell colonies.

### Western blot (WB) analysis

WB was used to detect the expression of paxillin and related proteins in cells. The specific methods have been described previously 24. The antibody information is shown in Supplementary Table S1.

### Cell transfection

GP-Transfer-mate was used to transfect siRNA and shRNA into cells. The pcDNA3.1 (+) vector was used to generate pcDNA3.1 (+) paxillin, Y118E, Y118F, and Y31F mutants, and the plasmid was transfected into cells using GP-Transfer-mate. The cell samples were collected after 48-72 h, and the transfection efficiency was assessed using WB. The siRNA and shRNA sequences are listed in supplementary Table S2-5.

### Co-immunoprecipitation

Co-immunoprecipitation was used to detect protein interactions. IP lysis buffer (Thermo Fisher Scientific) containing a mixture of hemiprotease and phosphatase inhibitor (1:100 dilution) was used to lyse cells on ice for 30 min. After centrifugation, the supernatant was collected and incubated with the antibodies overnight at 4 °C on a roller. Normal mouse immunoglobulin (Bevotime, Shanghai, China) was used as a negative control. The pre-washed protein A/G agarose beads (Thermo Fisher Scientific) were added to each IP tube and incubated at 4 °C for 2 hours. After washing and centrifugation, the IP results were confirmed by WB.

### ML141 and Y15 treatment

When confluency of the cells reached 60-80%, 20 μM ML141 or 2.5 μM, 5 μM Y15 was added, and the cells were collected after 24 h for subsequent experiments.

### Fluorescent dequenching (DQ) gelatin assay

250 μL 0.1 mg/mL DQ-gelatin (Life Technologies, Waltham, MA, USA) was added to a confocal dish and incubated overnight at 4 °C, followed by three washes with PBS. The cells were then added to pre-prepared DQ-gelatin-coated culture dishes. After incubation, fluorescein isothiocyanate (FITC) fluorescence produced by the cleavage of DQ-gelatin was observed using confocal microscopy.

### Human colorectal cancer (CRC) samples

Human paraffin-embedded CRC tissue specimens (n=182) were collected from the Department of Pathology of Tianjin Union Medical Center. These samples were categorized into four subgroups: 42 well-differentiated primary tumors (Group I), 52 moderately differentiated primary tumors (Group II), 51 poorly differentiated primary tumors (Group III), and 37 lymph node metastatic lesions (Group IV). This study was approved by the Hospital Review Board of Tianjin Union Medical Center, and the confidentiality of patient information was maintained. The ethical review batch number is (2024) Quick Review No. (B180), and the patients involved in this study have signed written informed consent forms.

### Immunohistochemical staining

After baking the tissue microarray for approximately 2-3 h, it was placed in xylene for dewaxing, followed by gradient alcohol hydration treatment, antigen thermal repair using citric acid buffer, and blocking with goat serum after adding endogenous peroxidase to block non-specific background staining. Paxillin antibody was added and incubated overnight at 4 °C. The next day, a biotin-labeled goat anti-mouse/rabbit IgG polymer and horseradish enzyme-labeled streptavidin working solution were added and incubated for 15 min. 3,3'-diaminobenzidine was used for color development. The nuclei were counterstained with hematoxylin. Staining was considered positive if the cytoplasm and nuclei were brownish-yellow.

### Scoring of IHC Staining

Image J software was used to quantify the positive cell ratio and staining intensity. The final score (staining index) was defined by the sum of positive cell ratio score and staining intensity score of each case. The positive cell ratio score was defined as follows: 0, <5%; 1, ≥5% and <30%; 2, ≥30% and <50%; 3, ≥50%. The staining intensity score was defined as follows: 0, no staining (negative); 1, faint yellow (weak); 2, brownish-yellow (moderate); 3, brown (strong). The staining index for each section was determined by the sum of the staining intensity and positive cell scores. Chi-square test was used to determine whether there were significant differences in paxillin staining indices between different groups.

### Statistical analysis

SPSS 16.0 (SPSS Inc., Chicago, IL, USA) was employed to process and analyze the complete dataset in this study. All histogram data were expressed as means ± standard deviations. The Kruskal-Wallis test was used to evaluate paxillin expression differences in CRC tissues. Other comparisons were performed using a two-tailed Student's t-test and Pearson's chi-square (χ^2^) test. Statistical significance was established at *P* < 0.05.

## Results

### PDCs have strong invasion, migration and proliferation ability and proteolytic ability

**Figure 1:**
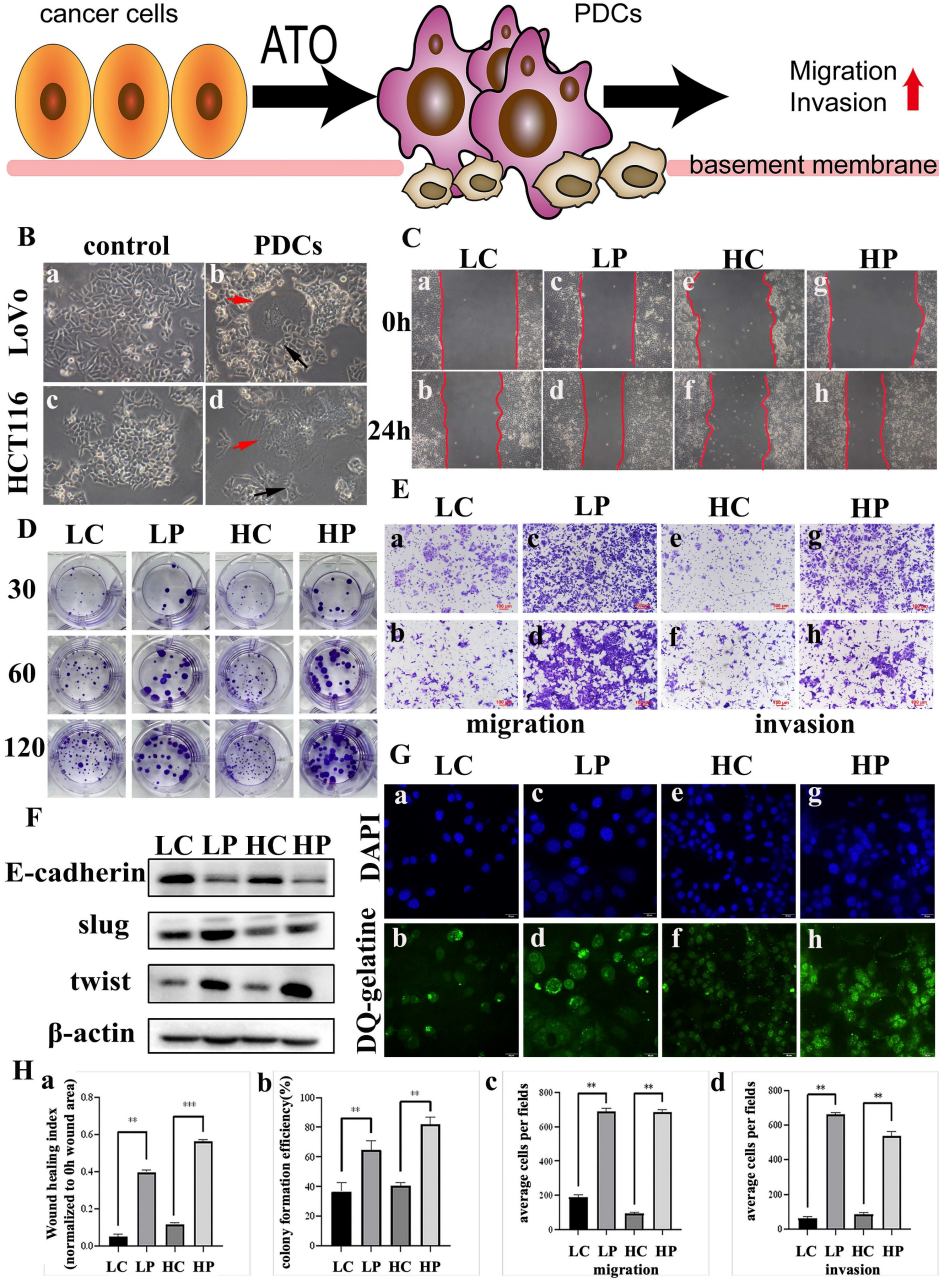
** (**A) Pattern: ATO induced ordinary diploid tumor cells to generate PGCCs with daughter cells with high invasion and migration ability. (B) PGCCs induction and daughter cells formation after treatment. (a) LoVo control cells; (b) LoVo PGCC (red arrow) and daughter cells (black arrow) after treatment; (c) HCT116 control cells; (d) HCT116 PGCC (red arrow) and daughter cells (black arrow) after treatment. (C) Wound healing in LoVo cells and HCT116 cells were measured before and after treatment with for 0 hours and 24 hours. (D) The colony formation of 30, 60 and 120 LoVo and HCT116 cells before and after treatment was determined. (E) Transwell migration and invasion assay of LoVo and HCT116 cells before and after treatment. (F) Western blot analysis of EMT-related protein expression (including E-cadherin, Slug and Twist) in LoVo and HCT116 cells before and after treatment. (G) FITC-DQ-gelatin was used to evaluate the proteolytic ability of LoVo cells and HCT116 cells before and after treatment. (H) (a) Statistical analysis of LoVo and HCT116 cell wound-healing assay before and after treatment. (b) Statistical analysis of LoVo and HCT116 plate cloning assay before and after treatment. (c) Statistical analysis of Transwell migration assay of LoVo and HCT116 before and after treatment. (d) Statistical analysis of Transwell invasion assay of LoVo and HCT116 before and after treatment. *P* value was calculated using one-way ANOVA. * P < 0.05; * * P < 0.01; *P* < 0.001. PGCCs: polyploid giant cancer cells, PDCs: PGCCs with daughter cells; LC: LoVo control cells; LP: LoVo PDCs; HC: HCT116 control cells; HP: HCT116 PDCs.

LoVo and HCT116 cell lines were treated with 32 μM ATO. After most of the cells died, the medium was replaced with fresh medium. This step was repeated at least three times. After cell recovery, multinucleated giant cells and mononuclear giant cell morphology appeared (Fig. 1B (b, d), red arrow). The surviving PGCCs produced PDCs by asymmetrical division (Fig. 1B (b, d), black arrow). The wound-healing assay showed that PDCs had a significantly higher migration ability (Fig. 1C; Fig. 1H (a)). PDCs had a significantly higher proliferation ability than control cells according to the plate colony-formation assay results (Fig. 1D; Fig. 1H (b)). Transwell migration and invasion assays showed that the PDC group had significantly higher migration and invasion abilities (Fig. 1A; Fig. 1E; Fig. 1H (c, d)). Using DQ-gelatin, we observed increased fluorescence in the PDCs in regions exhibiting gelatinolytic activity originating from the cellular surface (Fig. 1G). We detected epithelial-mesenchymal transition (EMT)-related proteins using WB. The levels of expression of epithelial markers such as E-cadherin in ATO-treated PDCs decreased, whereas the expression levels of transcription factors Slug and Twist increased significantly (Fig. 1F; Supplementary File 1: Fig. S1A (a-c)).

### CDC42 regulates the expression of integrin β1

CDC42 is a small GTPase associated with various human cancers. CDC42 exhibits a wide range of functions. Active CDC42 can modulate cell adhesion, cytoskeletal organization, and cell cycle progression by controlling the expression of proteins. Consequently, this regulation impacts cell proliferation, transformation, dynamic equilibrium as well as the invasive and migratory capabilities of tumor cells 25.

**Figure 2:**
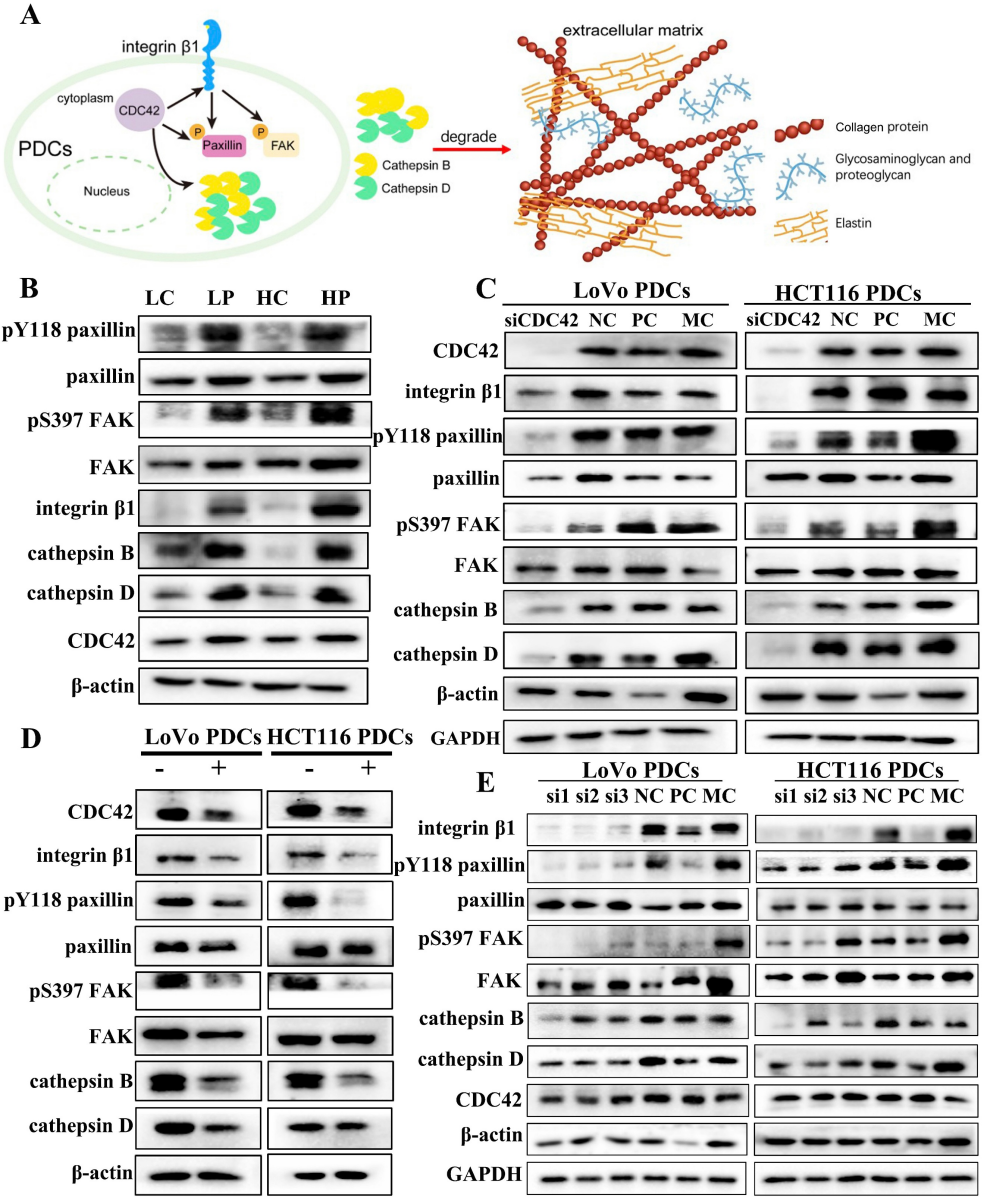
(A) Pattern: CDC42 regulates phosphorylation of paxillin to promote expression of cathepsin B/D. The brown beaded structure is collagen, the yellow structure is elastin, and the blue branched structure is glycosaminoglycan and proteoglycan. (B) Western blot results of Y118-paxillin, paxillin, S397-FAK, FAK, Integrin β1, cathepsin B/D and CDC42 protein expression in LoVo and HCT116 cells before and after treatment. (C) Western blot results of CDC42, integrin β1, Y118-paxillin, paxillin, S397-FAK, FAK and cathepsin B/D protein expression before and after transfection with siRNA-CDC42 in LoVo PDCs and HCT116 PDCs. (D) Western blot results of CDC42, integrin β1, Y118-paxillin, paxillin, S397-FAK, FAK and cathepsin B/D protein expression before and after ML141 intervention in LoVo PDCs and HCT116 PDCs. (E) Western blot results of integrin β1, Y118-paxillin, paxillin, S397-FAK, FAK, cathepsin B/D and CDC42 protein expression before and after transfection with siRNA- integrin β1 in LoVo PDCs and HCT116 PDCs.

Integrin β1 is a key mediator of signal transduction between ECM and cells. It plays a critical role during the onset and progression of tumors, including tumor initiation, initial invasion, and reactivation of metastatic potential in dormant disseminated tumor cells 26. CDC42 has been documented to accelerate cancer development through the activation of the integrin β1/FAK/paxillin signal transduction pathway 27. Cytoskeleton protein CDC42 and its downstream related proteins, including integrin β1, paxillin, FAK, Y118-paxillin, S397-FAK, and cathepsin B/D, were highly expressed in PDCs (Fig. 2B). The difference was statistically significant (Supplementary File 1: Fig. S1B). When siRNA was used to inhibit the expression of CDC42, the expression of integrin β1 decreased (Fig. 2C). Subsequently, cells were treated with ML141, a small-molecule inhibitor of CDC42, and the expression levels of related proteins were detected. After treatment with ML141, the expression level of integrin β1 decreased (Fig. 2D). This verified that CDC42 has the ability to modulate the expression of integrin β1.

### CDC42 and integrin β1 can regulate the phosphorylation of paxillin

Phosphorylation of paxillin can regulate the formation and turnover of focal adhesion, and paxillin phosphorylation at tyrosine residue 118 plays a crucial part in the development of catalytic complexes during cell-matrix adhesion 28. Y118-paxillin and S397-FAK were significantly inhibited, and the expression of cathepsin B/D was downregulated when siRNA was used to suppress the expression of CDC42 (Fig. 2C). Compared with the normal control (NC) group, the difference between the CDC42 knockdown groups exhibited statistical significance (Supplementary File 1: Fig. S1C, D). Subsequently, after treatment with ML141, the expression levels of Y118-paxillin, S397-FAK, and cathepsin B/D were downregulated (Supplementary File 1: Fig. S1E, F). Y118-paxillin and S397-FAK were significantly inhibited, and the expression of cathepsin B/D was downregulated when siRNA was used to knock down the expression of integrin β1. However, CDC42 expression did not change significantly, so we speculate that CDC42 affects paxillin by regulating integrin β1 expression (Fig. 2E). However, the regulatory relationship between paxillin and cathepsin B/D is unclear.

Cell functional experiments were conducted to assess the impact of CDC42 knockdown on PDC migration, invasion, and proliferation. In the wound-healing assay, the NC group exhibited narrower wound gaps than the CDC42 knockdown group (Supplementary File 1: Fig. S2A), and the difference reached statistical significance (Supplementary File 1: Fig. S2E (a)). The scratched areas of the PDCs before ML141 treatment were significantly narrower compared to those after ML141 treatment (Supplementary File 1: Fig. S2B). The plate colony-formation efficiency after CDC42 knockdown was significantly reduced in comparison to the NC group, and the plate colony-formation efficiency after ML141 treatment was noticeably decreased compared to that before ML141 treatment (Supplementary File 1: Fig. S2C, D).

### Phosphorylated paxillin can regulate cathepsin B/D

**Figure 3:**
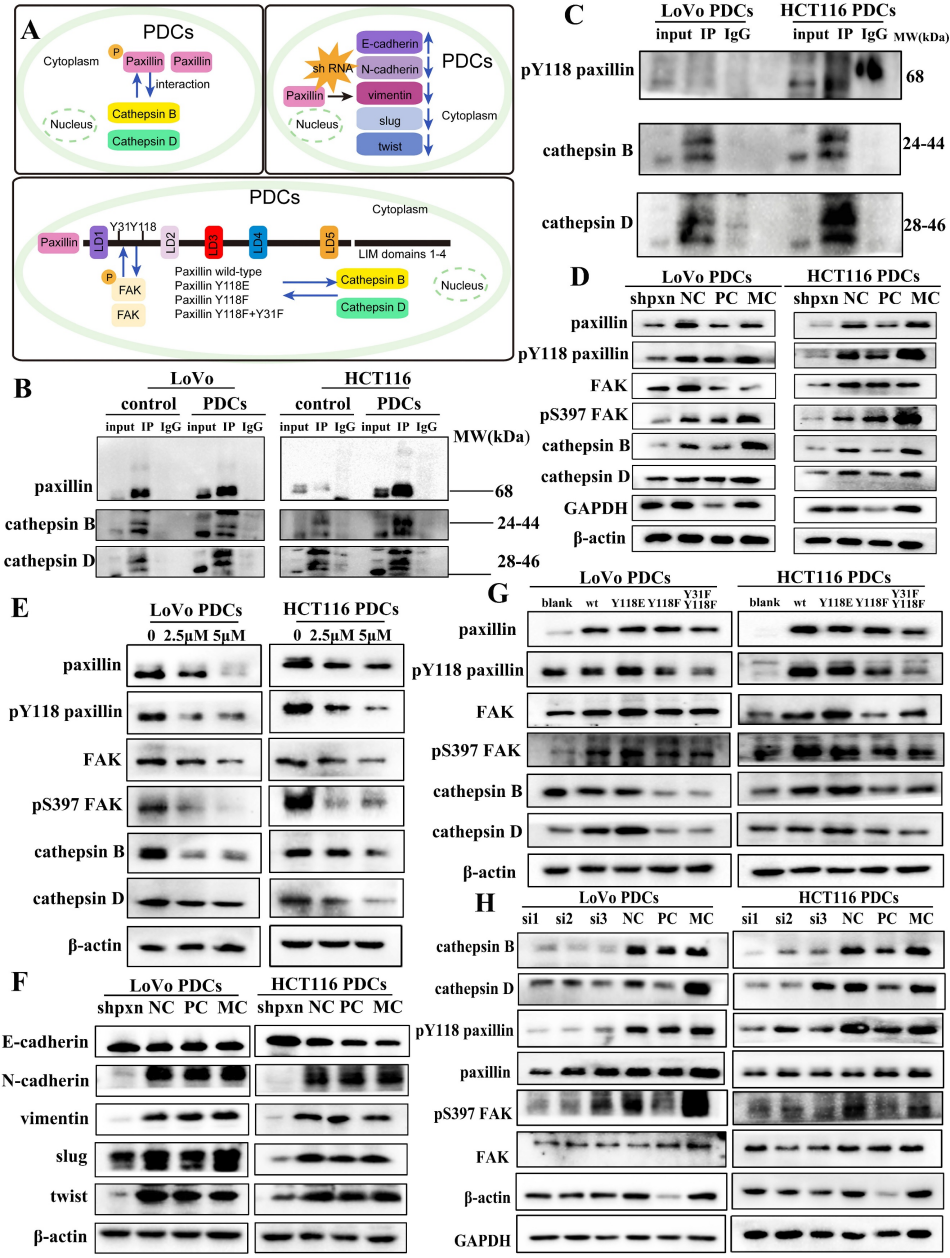
** (**A) Pattern (B) The results of paxillin co-immunoprecipitation (anti-paxillin antibody was used for immunoprecipitation). (C) The results of Y118-paxillin in LoVo PDCs and HCT116 PDCs (anti-Y118-paxillin antibody was used for immunoprecipitation). (D) Western blot results of paxillin, Y118-paxillin, FAK, S397-FAK and cathepsin B/D protein expression before and after transfection with shRNA-paxillin in LoVo PDCs and HCT116 PDCs. (E) Western blot results of paxillin, Y118-paxillin, FAK, S397-FAK and cathepsin B/D protein expression before and after Y15 intervention in LoVo PDCs and HCT116 PDCs. (F) The knockdown of PXN increased E-cadherin expression and decreased N-cadherin, vimentin, Slug and Twist expression in LoVo PDCs and HCT116 PDCs. (G) Western blot results of paxillin, Y118-paxillin, FAK, S397-FAK and cathepsin B/D protein expression before and after transfection with mutation of the paxillin site in LoVo PDCs and HCT116 PDCs. (H) Western blot results of cathepsin B/D, FAK, S397-FAK, paxillin and Y118-paxillin protein expression before and after transfection with siRNA-cathepsin B in LoVo PDCs and HCT116 PDCs.

To further study the activation mechanism of paxillin and its role in promoting higher invasion and migration ability, we used co-immunoprecipitation experiments and found that paxillin interacted with cathepsin B/D. Cathepsin B/D is a key member of the protease family that promotes tumor invasion, migration, and angiogenesis by degrading and remodeling the ECM. The role of cathepsin B/D-induced signal transduction in promoting tumorigenicity has been extensively studied. In this study, the co-immunoprecipitation results showed that cathepsin B/D interacted with paxillin (Fig. 3B) and with paxillin tyrosine 118 (Fig. 3C) in PDCs.

After knockdown of paxillin expression using shRNA, the expression of FAK and cathepsin B/D were significantly downregulated (Fig. 3D) compared with those of the NC group (Supplementary File 1: Fig. S3A, B). Subsequently, we used the small-molecule inhibitor Y15, an effective specific focal adhesion kinase inhibitor, to inhibit autophosphorylation and block the phosphorylation of the FAK downstream substrate paxillin. The expression of paxillin, Y118-paxillin, FAK, S397-FAK, and cathepsin B/D was downregulated after Y15 treatment (Fig. 3E). Their expression differed significantly between the non-Y15 treatment group and the Y15 treatment group (Supplementary File 1: Fig. S3C, D).

Numerous research studies have indicated that elevated phosphorylation of paxillin at residues Y31 and Y118 serves as an indicator of metastasis 29. Its phosphorylated state is typically associated with cancer cell metastasis. In order to ascertain the function of phosphorylated Y118-paxillin in cell migration, a pcDNA3.1 (+) vector was used to generate pcDNA3.1 (+) paxillin, Y118E, Y118F, and Y31F + Y118F, and the plasmids were transferred into cells using GP-Transfer-mate. WB experiments showed that compared with the WT group, the Y118E group exhibited an increase in cathepsin B/D expression, whereas the Y118F group displayed a decrease in cathepsin B/D expression. We mutated the Y31 and Y118 sites of paxillin into the non-phosphorylated state concurrently and found that the experimental results of the Y118F group were similar (Fig. 3G). We found that when the Y31 and Y118 of paxillin were altered, FAK expression was also affected. On the basis of the above findings, we deduce that changing the 118 and 31 sites of paxillin may affect its binding to FAK, thereby affecting the expression of downstream proteins. During cell migration, focal adhesions serve as macromolecular structures that create physical linkages between the cytoskeleton and the ECM. Focal adhesions play a crucial role in facilitating cell-cell adhesion and cell-ECM connections, with the quantity of these adhesions indicating the potential for cellular metastasis 30. We confirmed that these structures were located in the adhesion structures of PDCs using immunofluorescence (Supplementary File 1: Fig. S2F).

### Cathepsin B/D can also regulate phosphorylated paxillin

To determine whether cathepsin B/D promotes adhesion through paxillin signal transduction, we used siRNA to knockdown cathepsin B expression. The results of WB showed that after knockdown of cathepsin B expression, the expression of paxillin and FAK did not change significantly; however, the expression of phosphorylated paxillin, FAK, and cathepsin D were downregulated (Fig. 3H). This suggests that cathepsin B promotes the invasion and migration of PDCs by influencing cytoskeletal disturbances through the regulation of the activity of paxillin and FAK. The differences between the NC group and the siRNA-cathepsin B group were statistically significant (Supplementary File 1: Fig. S4A).

### Paxillin regulates the expression of markers of epithelial-mesenchymal transition (EMT)

Paxillin has been reported to play a role in EMT. We studied the mechanism by which paxillin promotes the invasion and migration of PDCs. After knockdown paxillin expression using shRNA in LoVo PDCs and HCT116 PDCs, the expression of EMT markers was evaluated using WB (Fig. 3F). The results showed that knocking down paxillin led to a reduction in the expression of two key mesenchymal markers, N-cadherin and vimentin, while simultaneously enhancing the expression of E-cadherin. Furthermore, the expression of Slug and Twist decreased. This indicates that paxillin downregulation inhibits the EMT process.

### Phosphorylated paxillin and cathepsin B/D participate in ECM degradation and promote the proliferation, migration and invasion of PDCs

**Figure 4:**
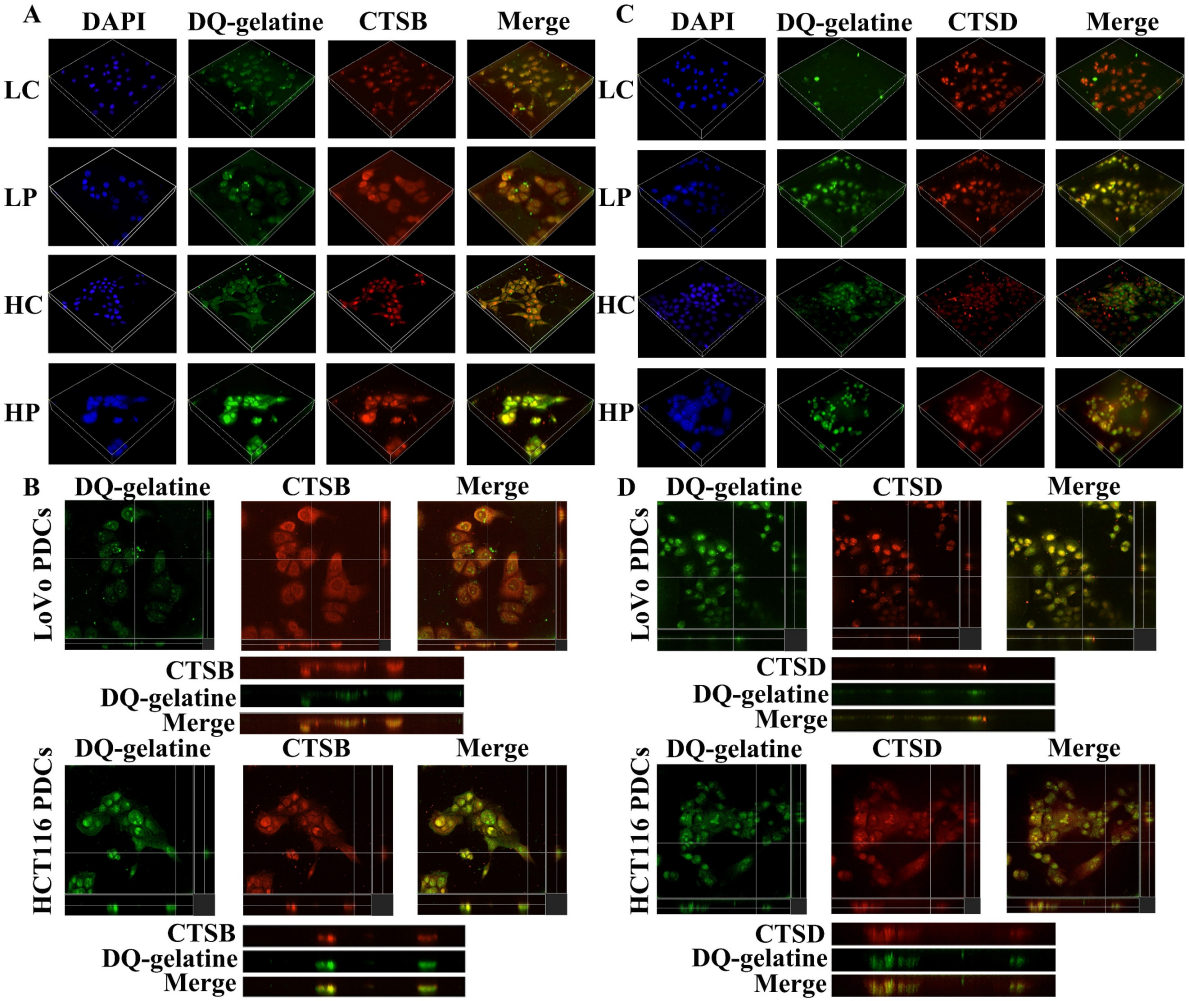
** (**A) 3D z-stacks of LoVo and HCT116 control group and PDCs group, observation of the localization of cathepsin B by confocal microscopy. (B) Observation of the co-localization of cathepsin B and degraded gelatin by YZ axis zoom. (C) 3D z-stacks of LoVo and HCT116 control group and PDCs group, observation of the localization of cathepsin D by confocal microscopy. (D) Observation of the co-localization of cathepsin D and degraded gelatin by YZ axis zoom.

Previous studies have shown that the PDC group has higher proteolytic ability than that of the control group. The gelatin degradation area is an area of invasive structure formation. Cathepsin B/D is involved in increasing the ability of ECM protein hydrolysis 31, 32. We used microscopy to examine whether cathepsin B/D colocalized with the gelatin degradation area by seeding cells on FITC-conjugated DQ-gelatin. The generated 3D z-stacks indicated that cathepsin B/D (red) mediates the degradation of gelatin (green). In addition, the co-localization of cathepsin B/D and the gelatin degradation area can be observed in the zoom of the YZ axis (Fig. 4).

**Figure 5:**
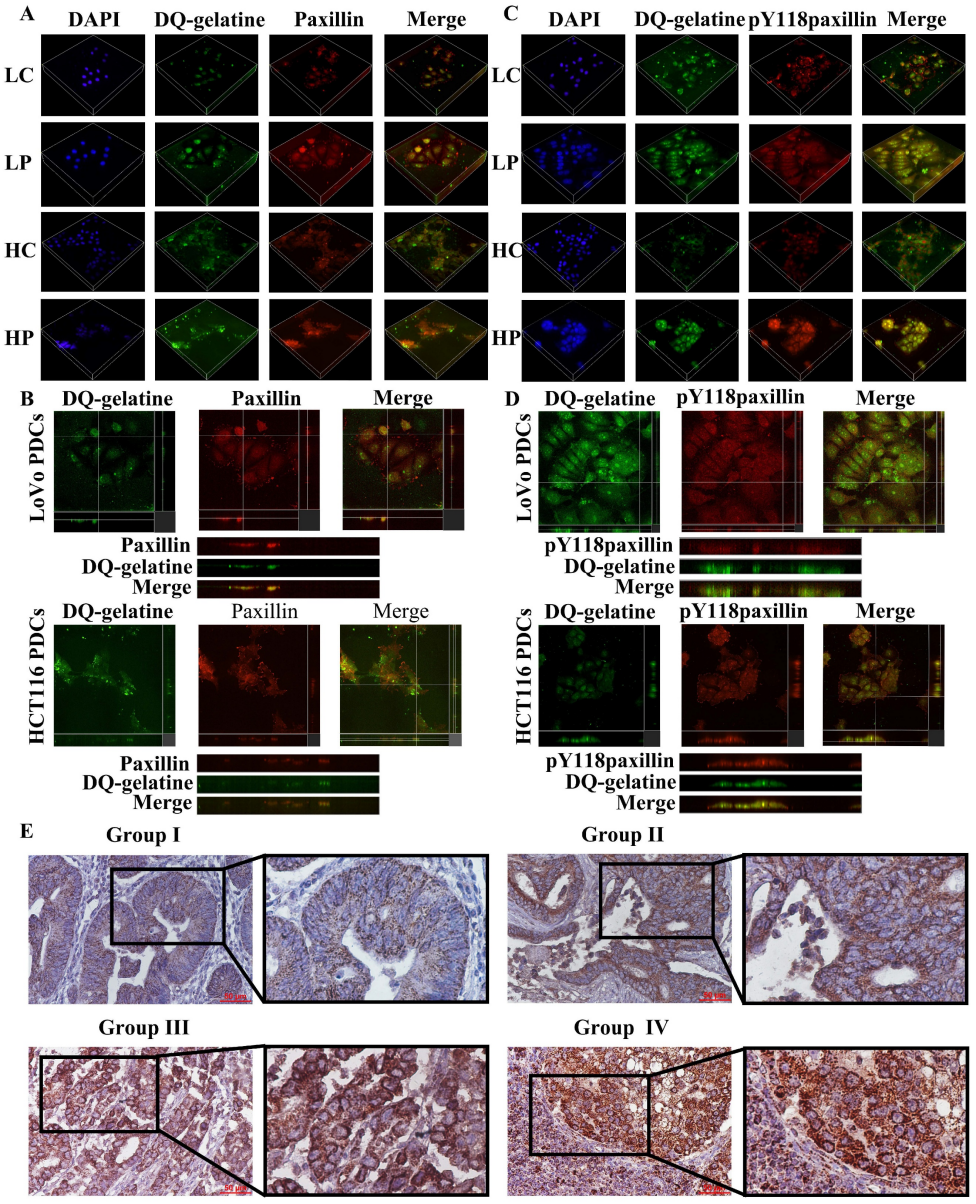
(A) 3D z-stacks of LoVo and HCT116 control group and PDCs group, observation of the localization of paxillin by confocal microscopy. (B) Observation of the co-localization of paxillin and degraded gelatin by YZ axis zoom. (C) 3D z-stacks of LoVo and HCT116 control group and PDC group, observation of the localization of Y118-paxillin by confocal microscopy. (D) Observation of the co-localization of Y118-paxillin and degraded gelatin by YZ axis zoom. (E) Immunohistochemical staining of paxillin in human colorectal cancer tissue. Group Ⅰ (well-differentiated CRC), Group Ⅱ (moderately differentiate CRC), Group Ⅲ (poorly differentiated CRC); Group Ⅳ (lymph node metastatic foci).

Paxillin is found in cell-matrix adhesion sites and invadopodia 33. In osteoclasts, tyrosine phosphorylation of paxillin promotes cell migration by participating in the ring expansion of invasive invadopodia 34. The LIM domain of paxillin is essential for the proteolytic activity of invadosomes, and all LD domains are capable of localizing paxillin in invadosomes and are sufficient for restoring their formation 35. Paxillin preferentially regulates invadosome assembly. To confirm that paxillin promotes the invasion and migration of PDCs by locating the invadosome and promoting the degradation of the ECM, we used the above method to inoculate cells on FITC-coupled DQ-gelatin. The generated 3D z-stacks and YZ zoom were used to visualize the localization of paxillin and Y118-paxillin in the invadosomes (Fig. 5). These results indicate that cathepsin B/D, paxillin, and their phosphorylation play a role in the degradation of the ECM by invadosomes (Fig. 2A). Targeting paxillin phosphorylation and cathepsin B/D could represent a promising approach for the treatment of metastatic cancer.

The role of paxillin and cathepsin B in promoting PDCs invasion, migration, and proliferation was determined using cell function experiments. Wound-healing assays showed that after paxillin knockdown, the migration ability of PDCs decreased (Supplementary File 1: Fig. S3E, F and J (a, b)), and after cathepsin B knockdown, the migration ability of PDCs also decreased (Supplementary File 1: Fig. S4B and E (a)). Plate cloning results showed that the proliferation ability of PDCs decreased after paxillin knockdown (Supplementary File 1: Fig. S3G, H and J (c, d)). Similarly, when the expression of cathepsin B decreased, the plate-cloning experiment showed that the proliferation ability of PDCs decreased (Supplementary File 1: Fig. S4C and E (b)). Transwell migration and invasion experiments confirmed that the migration and invasion ability of PDCs decreased after knockdown of paxillin by shRNA (Supplementary File 1: Fig. S3I and J (e, f)), and the migration and invasion ability of PDCs decreased after cathepsin B knockdown (Supplementary File 1: Fig. S4D and E (c, d)).

### Expression of paxillin in human CRC tissues

The expression level and clinicopathological significance of paxillin were evaluated in 182 human CRC tissue samples. The immunohistochemical evaluation demonstrated statistically significant differences in paxillin staining scores among the four study cohorts (*P* < 0.001; table 1). In addition, paxillin was predominantly localized within the cytoplasm of the tumor cells (Fig. 5E), and the paxillin staining index of poorly differentiated CRC was significantly higher than that of well-differentiated CRC (*P* < 0.001) and moderately differentiated CRC (*P* < 0.001). The paxillin staining index of lymph node metastases was also significantly elevated compared to that of well-differentiated (*P* < 0.001) and moderately differentiated CRC (*P* < 0.001) (Table 1).

## Discussion

Studies have shown that chemotherapy and radiotherapy can lead to cell dormancy 36, 37, and these surviving dormant cells can lead to cancer recurrence and diffuse metastasis. The formation of PGCCs can be induced by a hypoxic microenvironment, chemical reagents, radiotherapy, as well as Chinese herbal medications. After a certain period of incubation, dormant PGCCs can produce daughter cells through asymmetric division under favorable conditions 38. The role of PGCCs in chemoresistance, metastasis, and tumor recurrence, as well as their ability to modify the tumor microenvironment, make PGCCs a promising therapeutic target.

CDC42 is necessary for the assembly of ECM adhesion sites and the actin cytoskeleton is regulated by paxillin phosphorylation 39. Numerous studies have discovered that protein phosphorylation serves a crucial function in the regulation of focal adhesion formation and turnover 40, 41. Paxillin constitutes a significant element of focal adhesions and plays a key role in controlling cell adhesion and migration 42. Alterations in the expression levels, localization, or phosphorylation status of paxillin are frequently linked to the metastasis of cancer cells 43. Paxillin is phosphorylated under integrin-mediated cell-ECM adhesion 44, 45. Interactions may exist between the subsequent events triggered by paxillin tyrosine phosphorylation and the intracellular mechanisms regulating Rho GTPase activity 46. In CRC, cathepsin B/D levels often increase at the edge of tumor invasion and during tumor budding 47, 48, they are involved in cancer metastasis by altering ECM remodeling and promoting invasion. In our research, we verified that paxillin, CDC42, and cathepsin B/D were highly expressed in ATO-induced PGCCs and confirmed the role of paxillin, CDC42, and cathepsin B in conferring PDCs with higher invasion and migration ability using wound-healing, plate-clone formation, and transwell migration and invasion assays. We confirmed that CDC42 and integrin β1 promote the invasion and migration of PDCs by phosphorylating paxillin and FAK to regulate the expression of cathepsin B/D. Paxillin and cathepsin B/D regulate each other and promote the invasion and metastasis of PDCs by regulating the degradation of the ECM. In this study, we seeded the cells on FITC-conjugated DQ-gelatin, and fluorescence was detected in areas with gelatinolytic activity on the cell surface. The findings indicated that the PDCs had a stronger gelatin degradation ability than the control cells. Studies have found that invadosomes limit protease activity in cell areas in direct contact with the ECM, thereby accurately regulating cell invasion in vivo 49-51. Protease localization to invadosome structures promotes ECM degradation and remodeling. Previous reports have shown that cathepsins exist in invadosome structures and participate in cell movement 52. The influence of cathepsins on various aspects of tumor metastasis makes them attractive targets for cancer treatment. In cancer cells, cathepsin B localizes on the cellular surface, facilitating ECM degradation and orchestrating the protease cascade to guide the invasive front of metastatic cells. In PDCs, we noticed an accumulation of cathepsin B/D near the gelatin dissolution zone. Previous studies have shown that invadopodia dynamics are regulated by paxillin 34. Suppressing the interaction between FAK and paxillin in melanoma cells can inhibit invadopodia-mediated matrix degradation, thereby inhibiting cell invasion and metastasis 53. In this study, we showed the localization of paxillin and Y118-paxillin on invadosomes using a 3D z-stack and YZ zoom.

Paxillin's function and subcellular localization are rigorously controlled through phosphorylation processes. Increased phosphorylation of the Y31 and Y118 residues is considered a marker of metastasis. The phosphorylation of paxillin has been suggested to mediate the transduction of signals from cell adhesion to the reorganization of the cytoskeleton required for cell movement 54. To conduct a direct assessment of the role of Y118-paxillin, we performed site-directed mutagenesis studies and speculated that mutating the Y118 and Y31 sites of the paxillin may affect its binding to FAK, thereby affecting the expression of downstream proteins (cathepsin B/D). EMT activation is considered the most lethal feature of tumor invasion, metastasis, and chemotherapy resistance. In PDCs, the expression of the transcription factors Slug and Twist, which control EMT, were higher than those of the control group. Knockdown of paxillin expression resulted in decreased N-cadherin and vimentin expression, increased E-cadherin expression, and significantly decreased Slug and Twist expression. This indicates that paxillin plays a role in the process of EMT in PDCs (Fig. 3A).

## Conclusion

Our study showed that CRC cells treated with ATO produced PDCs with high invasion and migration ability, which is consistent with the poor clinical efficacy of ATO in the treatment of solid tumors. In addition, our study suggests that the interaction between CDC42, paxillin, and cathepsin B enhances the invasion, migration, and proliferation of PDCs. Targeted therapies to inhibit their expression may provide novel strategies for reducing tumor recurrence and metastasis.

## Supplementary Material

Supplementary figures and tables.

## Figures and Tables

**Table 1:** Differences of paxillin staining index in different group of human colorectal cancers

	Group	N	Staining index for paxillin	Value of statistics	P
Well-differentiated CRC	Group I	42	6.295±2.488	χ^2^ = 41.245	<0.001*
Moderately differentiated CRC	Group II	51	6.708 ± 2.193		
Poorly differentiated CRC	Group III	52	9.849 ± 1.932		
Lymph node metastatic foci	Group IV	37	10.020 ± 1.450		

∗*P* < 0.05: statistically significant. *P*: difference among the group; *P1:* difference between groups I and II =0.395; *P2:* difference between groups II and III) < 0.001; *P3:* difference between groups III and IV = 0.653; *P4:* difference between groups I and IV < 0.001; *P5:* difference between groups II and IV < 0.001. CRC: colorectal cancer.

## Abbreviations

PGCCs: Polyploid giant cancer cells
PDCs: PGCCs with daughter cells
CRC: colorectal cancer
DQ: Dequenching
ATO: arsenic trioxide
CDC42: cell division cycle 42
WB: western blot
EMT: epithelial-mesenchymal transition
ECM: extracellular matrix
