# Supplementary material for: Paxillin promotes the invasion and migration of polyploid giant cancer cells with daughter cells after arsenic trioxide treatment by regulating the expression of cathepsin B/D
Source: J Cancer. 2025 Jul 24;16(11):3329–42. doi: 10.7150/jca.115892 (PMC12374962; doi:10.7150/jca.115892)
Supplement: Supplementary file 1 — Supplementary figures and tables. [file jcav16p3329s1.pdf]

Supplementary Table 1: Detail information of antibodies used in the paper.

Antibody	Species specificity	Company	WB	IF
CDC42	Rabbit monoclonal	Abcam	1:1000	1:100
cathepsin B	Rabbit monoclonal	CST	1:1000	1:100
cathepsin D	Rabbit polyclonal	Proteintech	1:1000	1:100
paxillin	Rabbit monoclonal	CST	1:1000	1:100
paxillin tyr-118	Rabbit monoclonal	CST	1:1000	1:100
FAK	Mouse monoclonal	Proteintech	1:1000	
FAK ser-397	Rabbit polyclonal	Affinity	1:1000	
E-cadherin	Rabbit polyclonal	Proteintech	1:1000	
twist	Rabbit polyclonal	Proteintech	1:1000	
integrin β1	Rabbit polyclonal	Proteintech	1:1000	
slug	Mouse monoclonal	Santa	1:500	
β-actin	Mouse monoclonal	Proteintech	1:2000	
GAPDH	Rabbit polyclonal	Affinity	1:2000	

Supplementary Table 2 CDC42-siRNA interfering sequences.

siRNA	Sense （5’-3’）	Antisense （5’-3’）
CDC42-homo-532	CCUCUACUAUUGAGAAACUTT	AGUUUCUCAAUAGUAGAGGTT
CDC42-negative control	UUCUCCGAACGUGUCACGUTT	ACGUGACACGUUCGGAGAATT
CDC42-β-actin	UGAAGAUCAAGAUCAUUGCTT	GCAAUGAUCUUGAUCUUCATT

Supplementary Table 3 paxillin-shRNA interfering sequences.

shRNA	Sense （5’-3’）
paxillin-homo	GGGCAGCAACCTTTCTGAACT
paxillin-negative control	GTTCTCCGAACGTGTCACGT
paxillin- GAPDH	TATGACAACAGCCCTCAAG

Supplementary Table 4 Cathepsin B-siRNA interfering sequences.

siRNA	Sense （5’-3’）	Antisense （5’-3’）
cathepsin B-homo-1	GCUGGUCAACUAUGUCAACAATT	UUGUUGACAUAGUUGACCAGCTT
cathepsin B-homo-2	UCGGAUGAGCUGGUCAACUAUTT	AUAGUUGACCAGCUCAUCCGATT
cathepsin B-homo-3	CGACCUACAAACAGGACAATT	UUGUCCUGUUUGUAGGUCGTT
cathepsin B- negative control	UGACCUCAACUACAUGGUUTT	AACCAUGUAGUUGAGGUCATT
Cathepsin B-β-actin	UUCUCCGAACGUGUCACGUTT	ACGUGACACGUUCGGAGAATT

Supplementary Table 5 Integrin β1-siRNA interfering sequences.

siRNA	Sense （5’-3’）	Antisense （5’-3’）
integrin β1-homo-1	CUGUUCUUUGGAUACUAGUTT	ACUAGUAUCCAAAGAACAGTT
integrin β1-homo-2	GCCCUCCAGAUGACAUAGAAATT	UUUCUAUGUCAUCUGGAGGGCTT
integrin β1-homo-3	CCGUAGCAAAGGAACAGCATT	UGCUGUUCCUUUGCUACGGTT
integrin β1- negative control	UGACCUCAACUACAUGGUUTT	AACCAUGUAGUUGAGGUCATT
integrin β1-β-actin	UUCUCCGAACGUGUCACGUTT	ACGUGACACGUUCGGAGAATT

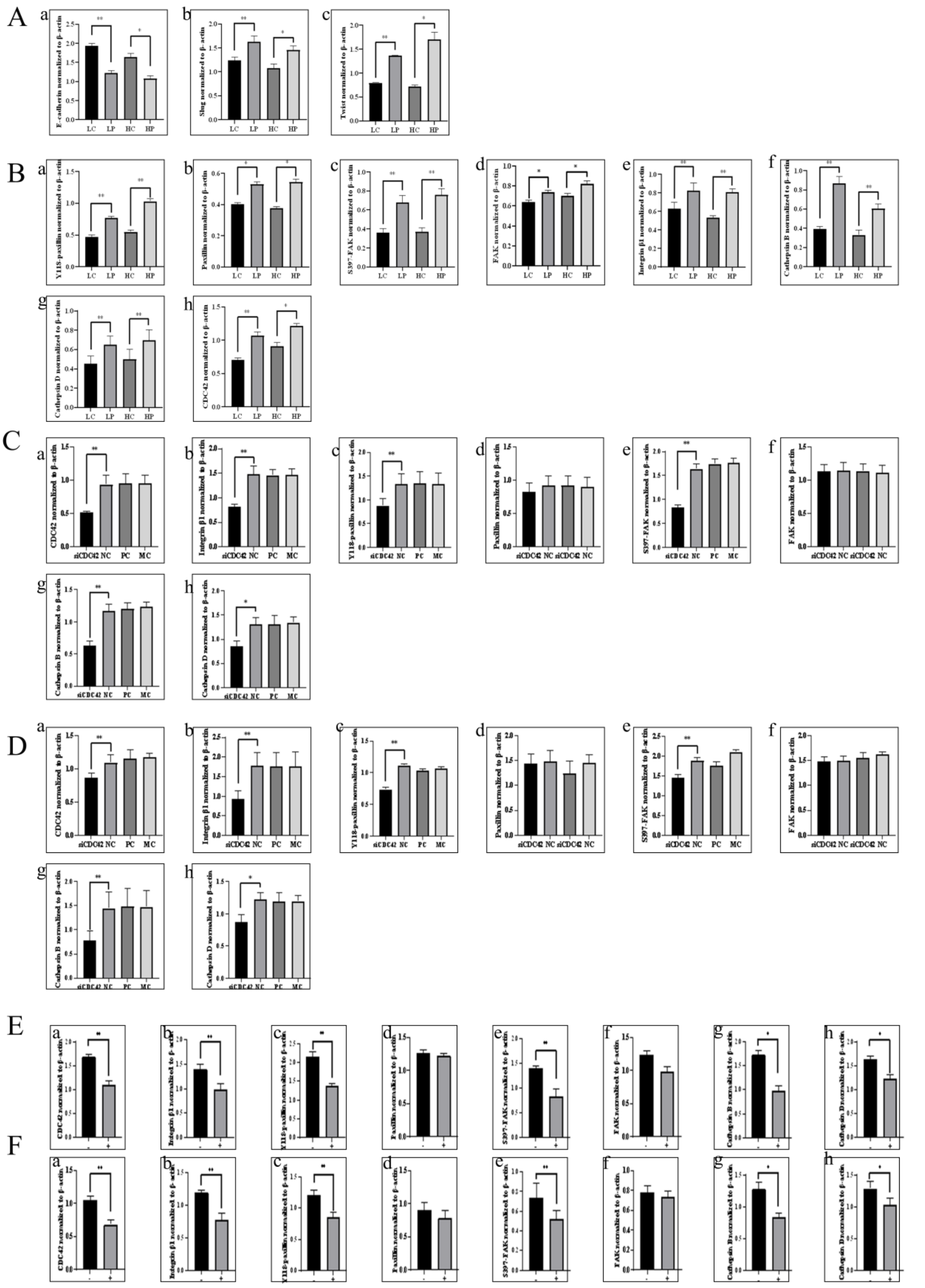

Figure S1. Gray value analysis of western blots. All data represent the means ± standard errors of means of at least three independent experiments. (A) (a) E-cadherin, (b) Slug and (c) Twist expression in LoVo and HCT116 cells with and without ATO treatment. (B) (a) Y118-paxillin, (b) paxillin and (c) s397-FAK, (d) FAK, (e) integrin

β1, (f, g) cathepsin B/D and (h)CDC42 expression in LoVo and HCT116 cells with and without ATO treatment. (C) (a) CDC42, (b) integrin β1, (c) Y118-paxillin, (d) paxillin and (e) s397-FAK, (f) FAK and (g, h) cathepsin B/D expression in LoVo PDCs before and after siRNA-CDC42. (D) (a) CDC42, (b) integrin β1, (c) Y118-paxillin, (d) paxillin and (e) s397-FAK, (f) FAK, (g, h) cathepsin B/D expression in HCT116 PDCs before and after siRNA-CDC42. (E) (a) CDC42, (b) integrin β1, (c) Y118-paxillin, (d) paxillin and (e) s397-FAK, (f) FAK, (g, h) cathepsin B/D expression before and after ML141 intervention in LoVo PDCs. (F) (a) CDC42, (b) integrin β1, (c) Y118-paxillin, (d) paxillin and (e) s397-FAK, (f) FAK, (g, h) cathepsin B/D expression before and after ML141 intervention in LoVo PDCs.

P values are calculated using the one-way analysis of variance. *, $P < 0.05$; **, $P < 0.01$; ***, $P < 0.001$.

PDCs: PGCCs with daughter cells; ATO: arsenic trioxide; LC: LoVo control cells; LP: LoVo PDCs after ATO treatment; HC: HCT116 control cells; HP: HCT116 PDCs after ATO treatment.

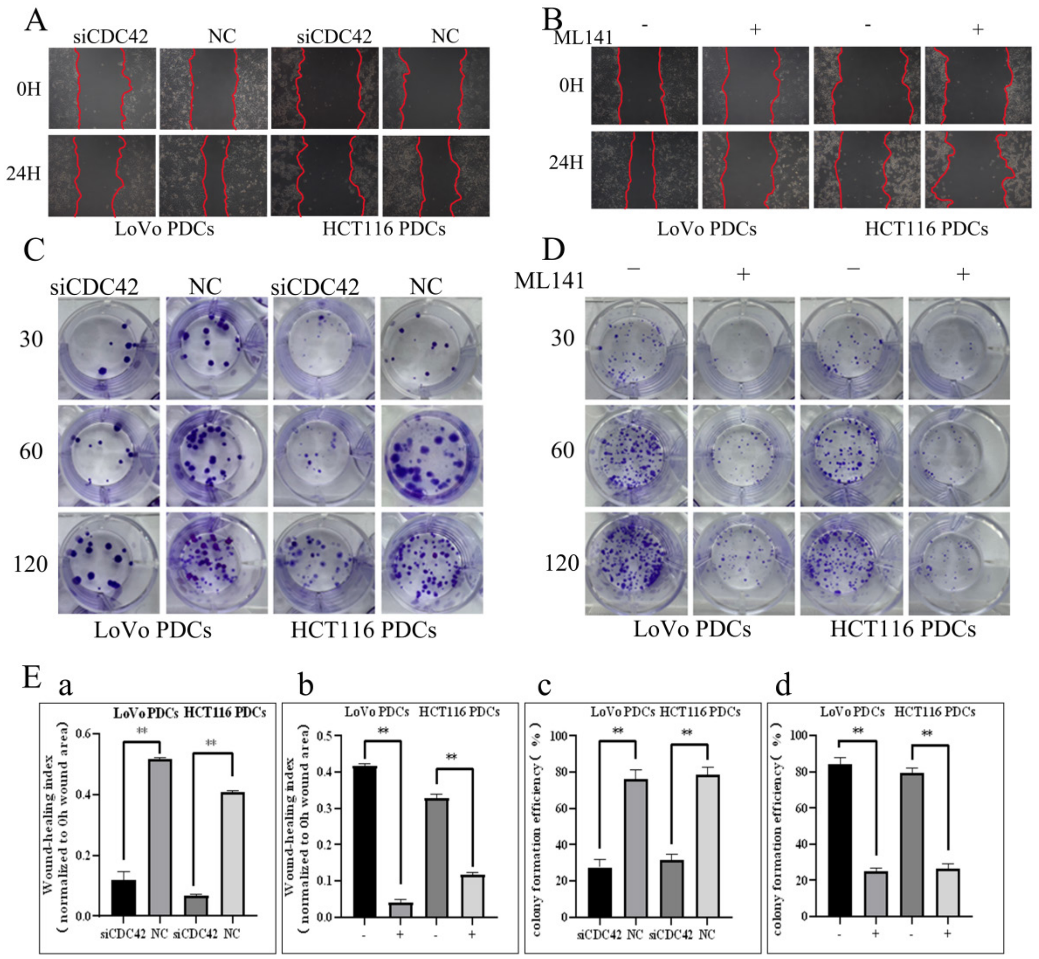

Figure S2. **(**A) Wound healing in LoVo PDCs and HCT116 PDCs were measured before and after transfection with siRNA-CDC42 at 0 h and 24 h. (B) Wound healing in LoVo PDCs and HCT116 PDCs were measured before and after ML141 intervention at 0 h and 24 h. (C) Colony formation of 30, 60 and 120 LoVo PDCs and HCT116 PDCs before and after transfection with siRNA-CDC42. (D) Colony formation of 30, 60 and 120 LoVo PDCs and HCT116 PDCs before and after ML141 intervention. (E) (a) Statistical analysis of the wound-healing index of LoVo PDCs and HCT116 PDCs before and after transfection with siRNA-CDC42. (b) Statistical analysis of the wound-healing index of LoVo PDCs and HCT116 PDCs before and after ML141 intervention. (c) Differences in colony-formation efficiency of LoVo PDCs and HCT116 PDCs before and after transfection with siRNA-CDC42. (d) Differences in colony-formation efficiency of LoVo PDCs and HCT116 PDCs before and after ML141 intervention.

P values are calculated using the one-way analysis of variance. *, $P < 0.05$;

**, $P < 0.01$; ***, $P < 0.001$.

PDCs: PGCCs with daughter cells; ATO: arsenic trioxide; LC: LoVo control cells; LP: LoVo PDCs after ATO treatment; HC: HCT116 control cells; HP: HCT116 PDCs after ATO treatment.

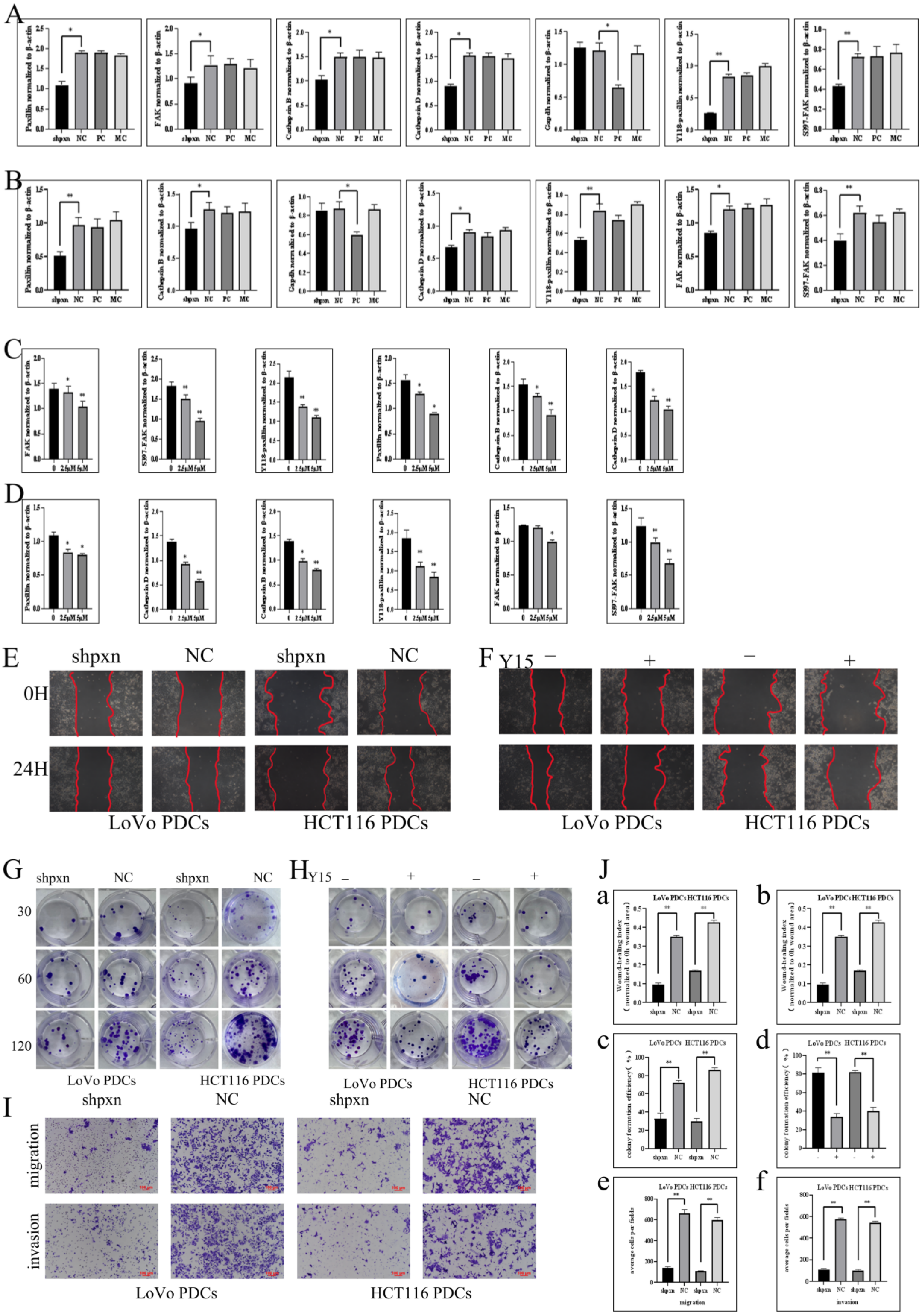

Figure S3. (A) Gray value analysis of western blots. paxillin, Y118-paxillin, FAK, s397-FAK and cathepsin B/D expression in LoVo PDCs before and after shRNA-paxillin. (B) paxillin, Y118-paxillin, FAK, s397-FAK and cathepsin B/D expression in

HCT116 PDCs s before and after shRNA-paxillin. (C) paxillin, Y118-paxillin, FAK, s397-FAK and cathepsin B/D expression in LoVo PDCs before and after Y15 intervention. (D) paxillin, Y118-paxillin, FAK, s397-FAK and cathepsin B/D expression in HCT116 PDCs before and after Y15 intervention. (E) Wound healing in LoVo PDCs and HCT116 PDCs were measured before and after transfection with shRNA-paxillin at 0 h and 24 h. (F) Wound healing in LoVo PDCs and HCT116 PDCs were measured before and after Y15 intervention at 0 h and 24 h. (G) Colony formation of 30, 60 and 120 LoVo PDCs and HCT116 PDCs before and after transfection with shRNA-paxillin. (H) Colony formation of 30, 60 and 120 LoVo PDCs and HCT116 PDCs before and after Y15 intervention. (I) Transwell migration and invasion LoVo PDCs and HCT116 PDCs before and after transfection with shRNA-paxillin. (J) (a) Statistical analysis of the wound-healing index of LoVo PDCs and HCT116 PDCs before and after transfection with shRNA-paxillin. (b) Statistical analysis of the wound-healing index of LoVo PDCs and HCT116 PDCs before and after Y15 intervention. (c) The differences in colony-formation efficiency of LoVo PDCs and HCT116 PDCs before and after transfection with shRNA-paxillin. (d) The differences in colony-formation efficiency of LoVo PDCs and HCT116 PDCs before and after Y15 intervention. (e, f) Comparison of the average cell number in invasion and migration assay of LoVo PDCs and HCT116 PDCs before and after transfection with shRNA-paxillin.

P values are calculated using the one-way analysis of variance. *, $P < 0.05$; **, $P < 0.01$; ***, $P < 0.001$.

PDCs: PGCCs with daughter cells; ATO, arsenic trioxide; LC, LoVo control cells; LP, LoVo PDCs after ATO treatment; HC, HCT116 control cells; HP, HCT116 PDCs after ATO treatment; paxillin, pxn.

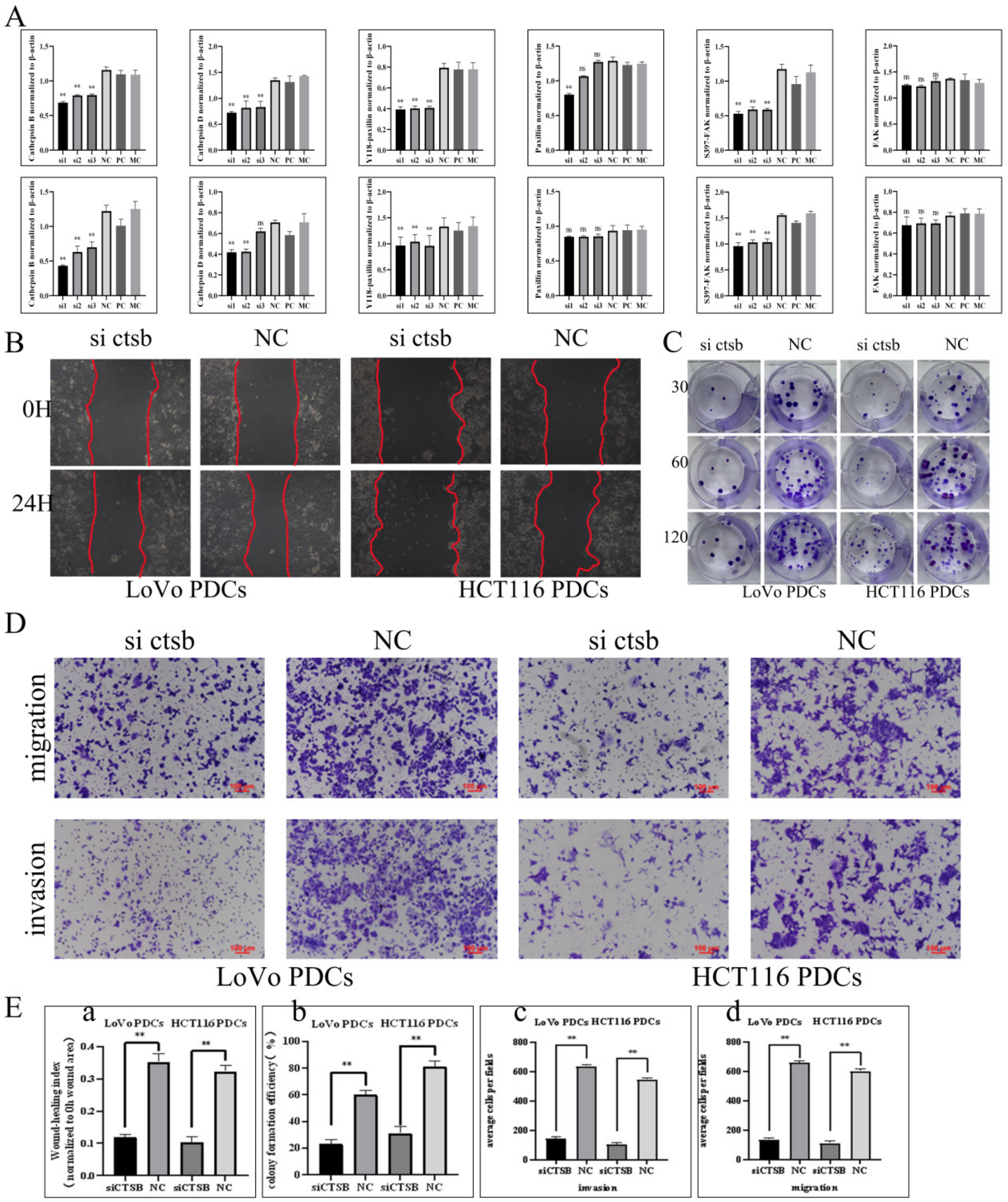

Figure S4. (A) Gray value analysis of western blots. cathepsin B/D, Y118-paxillin, paxillin, s397-FAK and FAK expression in LoVo PDCs and HCT116PDCs before and after siRNA-cathepsin B. (B) Wound healing in LoVo PDCs and HCT116 PDCs were measured before and after transfection with siRNA-cathepsin B at 0 h and 24 h. (C) Colony formation of 30, 60 and 120 LoVo PDCs and HCT116 PDCs before and after transfection with siRNA-cathepsin B. (D) Transwell migration and invasion LoVo PDCs and HCT116 PDCs before and after transfection with siRNA-cathepsin B. (E) (a)

Statistical analysis of the wound-healing index of LoVo PDCs and HCT116 PDCs before and after transfection with siRNA-cathepsin B. (b) Differences in colony-formation efficiency of LoVo PDCs and HCT116 PDCs before and after transfection with siRNA-cathepsin B. (c, d) Comparison of the average cell number in invasion and migration assay of LoVo PDCs and HCT116 PDCs before and after transfection with siRNA-cathepsin B.

P values are calculated using the one-way analysis of variance. *, $P < 0.05$; **, $P < 0.01$; ***, $P < 0.001$.

PDCs: PGCCs with daughter cells; ATO, arsenic trioxide; LC, LoVo control cells; LP, LoVo PDCs after ATO treatment; HC, HCT116 control cells; HP, HCT116 PDCs after ATO treatment; cathepsin B, ctsb.
